# Transgenerational effects from single larval exposure to azadirachtin on life history and behavior traits of *Drosophila melanogaster*

**DOI:** 10.1038/s41598-019-53474-x

**Published:** 2019-11-19

**Authors:** M. Ferdenache, R. Bezzar-Bendjazia, F. Marion-Poll, S. Kilani-Morakchi

**Affiliations:** 1 Laboratory of Applied Animal Biology, Department of Biology, Faculty of Sciences, Badji Mokhtar University of Annaba, 23000 Annaba, Algeria; 2 Evolution, Génomes, Comportement, Ecologie. CNRS, IRD, Univ Paris-Sud. Université Paris-Saclay, F-91198 Gif-sur-Yvette, France; 3 AgroParisTech, Paris, France

**Keywords:** Physiology, Environmental sciences

## Abstract

Azadirachtin is one of the successful botanical pesticides in agricultural use with a broad-spectrum insecticide activity, but its possible transgenerational effects have not been under much scrutiny. The effects of sublethal doses of azadirachtin on life-table traits and oviposition behaviour of a model organism in toxicological studies, *D. melanogaster*, were evaluated. The fecundity and oviposition preference of flies surviving to single azadirachtin-treated larvae of parental generation was adversely affected and resulted in the reduction of the number of eggs laid and increased aversion to this compound over two successive generations. In parental generation, early exposure to azadirachtin affects adult’s development by reducing the number of organisms, delay larval and pupal development; male biased sex ratio and induced morphological alterations. Moreover, adult’s survival of the two generations was significantly decreased as compared to the control. Therefore, Single preimaginal azadirachtin treatment can affect flies population dynamics *via* transgenerational reductions in survival and reproduction capacity as well as reinforcement of oviposition avoidance which can contribute as repellent strategies in integrated pest management programs. The transgenerational effects observed suggest a possible reduction both in application frequency and total amount of pesticide used, would help in reducing both control costs and possible ecotoxicological risks.

## Introduction

The effect of insecticides and other toxicants on insects have been traditionally assessed using measures of the acute mortality as a single endpoint and have relied on the determination of the acute lethal dose/concentration^1^. However, in addition to the direct effect on lethality these compounds may also impair various key biological traits of the individuals that survive exposure through physiological and behavioral effects^1,2^. Among physiological effects, developmental success, morphological deformities, adult longevity, sex ratio, fertility and fecundity are commonly estimated^3,4^. Behavioral effects may be manifested as impairment in insect mobility, learning ability, host finding, sexual communication as well as feeding and oviposition behavior^5–10^. An accurate assessment of these effects is crucial to acquire knowledge on the overall insecticide efficacy for long-term management of pest insect populations, as well as on their selectivity toward non-target species^11^. Indeed, when studying susceptibility of organisms towards insecticides, and beside the short term influences on the directly exposed individuals, it is important to take into account the entire life-history as a comprehensive method for evaluating the total effect on insect population, including the impacts on the next generation which have important implications for the success of an Integrated Pest Management (IPM) program^2,4^.

Among nowadays the insecticides used in sublethal effect studies, the botanical insecticides have been the subject of an increasing number of academic research as a potential option for an environment friendly pest management tools^12,13^ due to their rapid degradation in the environment, low mammalian toxicity, low risk of resistance development in target pest populations and good selectivity to non-target arthropods^14–18^. Azadirachtin (AZA), a natural tetranortriterpenoid compound extracted from the neem tree, *Azadirachta indica*^19^, is considered as one of the most promising plant compounds for pest control in organic agriculture^14,20^. AZA shows variable effects on insects including the model insect *Drosophila melanogaster*^21,22^. This triterpenoid acts as sterilant, insect growth regulators by disruption of the endocrine system, repellent, oviposition and feeding deterrent by activating bitter sensitive gustatory cells^23,24^. Larval exposure of *D. melanogaster* to sublethal doses of azadirachtin was found to affects various aspects of their physiology including digestive enzymes^25^ and this effect is also further observed in the adults^10^. This pre-imaginal exposure affects not only the physiology and the fitness of flies but also adults oviposition and feeding preference^7,10^.

Most studies on sublethal effects of insecticides are related to continuously or repeated exposure. This exposure provokes a generalized stress and activating a detoxification response such as up-regulated of cytochrome P450 genes which might lead to the detoxification of insecticide and even the development of resistance^26^. Moreover, the up-regulation is thought to provide versatility in environmental adaptation^27^. In botanical insecticide the potential fast desensitization to a feeding deterrent was reported^28,29^. Individual insects initially deterred by feeding inhibitor become increasingly tolerant due to repeated or continuous exposure^29^. Bomford and Isman^15^ reported an habitation to pure azadirachtin in the tobacco cutworms which become less sensitive to the antifeedant properties of azadirachtin, but not to a neem containing a same absolute amount of azadirachtin. This might have an important implication to avoid desensitization to commercial neem-based insecticides which contains additional non AZA-compounds^15^. Larval exposure to Neem Azal, a commercial Azadirachtin-rich based formulation, was found to enhance avoidances of this compound in adults of *D. melanogaster* surviving from previously treated larvae^10,25^. This long-lasting avoidance is related to conditioned aversion and may be related to another mechanism such as sensitization^30,31^ which also generally occurs after long term or repeated exposure and may increase avoidance to noxious stimulus^32^. Moreover, increasing evidence has highlighted the critical role of early life experience in adult physiology and behavior in insect^33^. Recent studies have revealed that insect can modulate their behavior on the basis of previous experiences early life and that various insecticide-mediated changes in the directly exposed generation can persist into the subsequent non-exposed generations^34,35^. Previously, we have focused on the impact of larval exposure to azadirachtin on adult’s fitness (fecundity, survival) and oviposition site preference of the parental generation of *D. melanogaster* as a model organism for testing insecticide activity^7^. Current study aimed to evaluate, the possible adverse effects of this prior single exposure to azadirachtin experienced by the preceding generations on life table and oviposition site preference of the filial generations. We monitored the oviposition site preference, fecundity, development, sex ratio, survival and morphological abnormalities of exposed and non-exposed generations. All these parameters were investigated over generations until their restoration to predict the outcome of azadirachtin use on pest management practices.

## Results

### Fecundity and oviposition site preference

**Figure 1:**
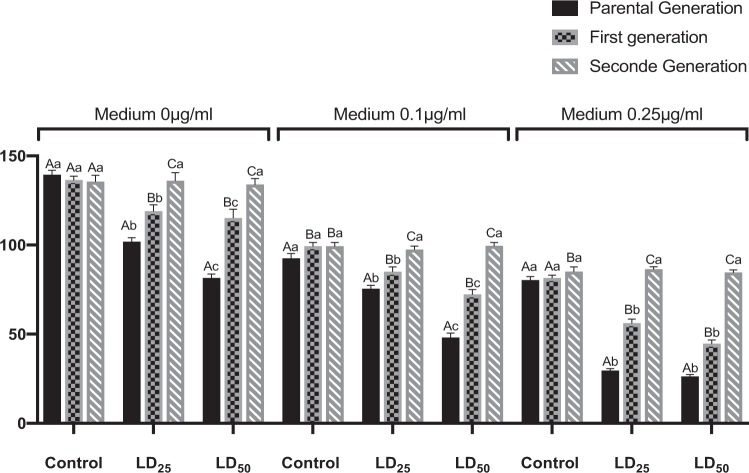
Effect of azadirachtin (LD_25_ and LD_50_), topically applied on early third instars larvae of *D. melanogaster* on fecundity of females (number of eggs laid) subjected to non-choice experiments (m ± SE; n = 12 replicates of 3 flies). Different small letters indicate a significant difference between control and treated individuals of the same medium (P < 0.05). Different capital letters indicate a significant difference between generations of the same medium (P < 0.05).

Azadirachtin, topically applied on the 3^rd^ instar larvae (LD_25_ and LD_50_ of immature stages) affect fecundity of females by a significant reduction of the number of eggs laid as compared to controls (KW = 24.73; p < 0.001). This reduction was observed over two successive generations (parental and F1), however, the total eggs laid was higher in the unexposed generation (F1) than in parental (P) ones (KW = 50.89; p < 0.001) (Fig. 1). Full restoration of affected fecundity was noted in the second generations (F2).

Results of oviposition preference in the no choice experiments (Fig. 1) revealed a clear preference for oviposition on untreated medium than in azadirachtin-treated ones.

For parental generation, Kruskal-Wallis test revealed significant effects in medium 0.1 μg/ml (KW = 29.42; p < 0.001) and medium 0.25 μg/ml (KW = 24.73; p < 0.001). In the first generation, a significant effect was also noted for medium 0.1 μg/ml (KW = 22.95; p < 0.001) and medium 0.25 μg/ml (KW = 27, 93; p < 0.001).

**Figure 2:**
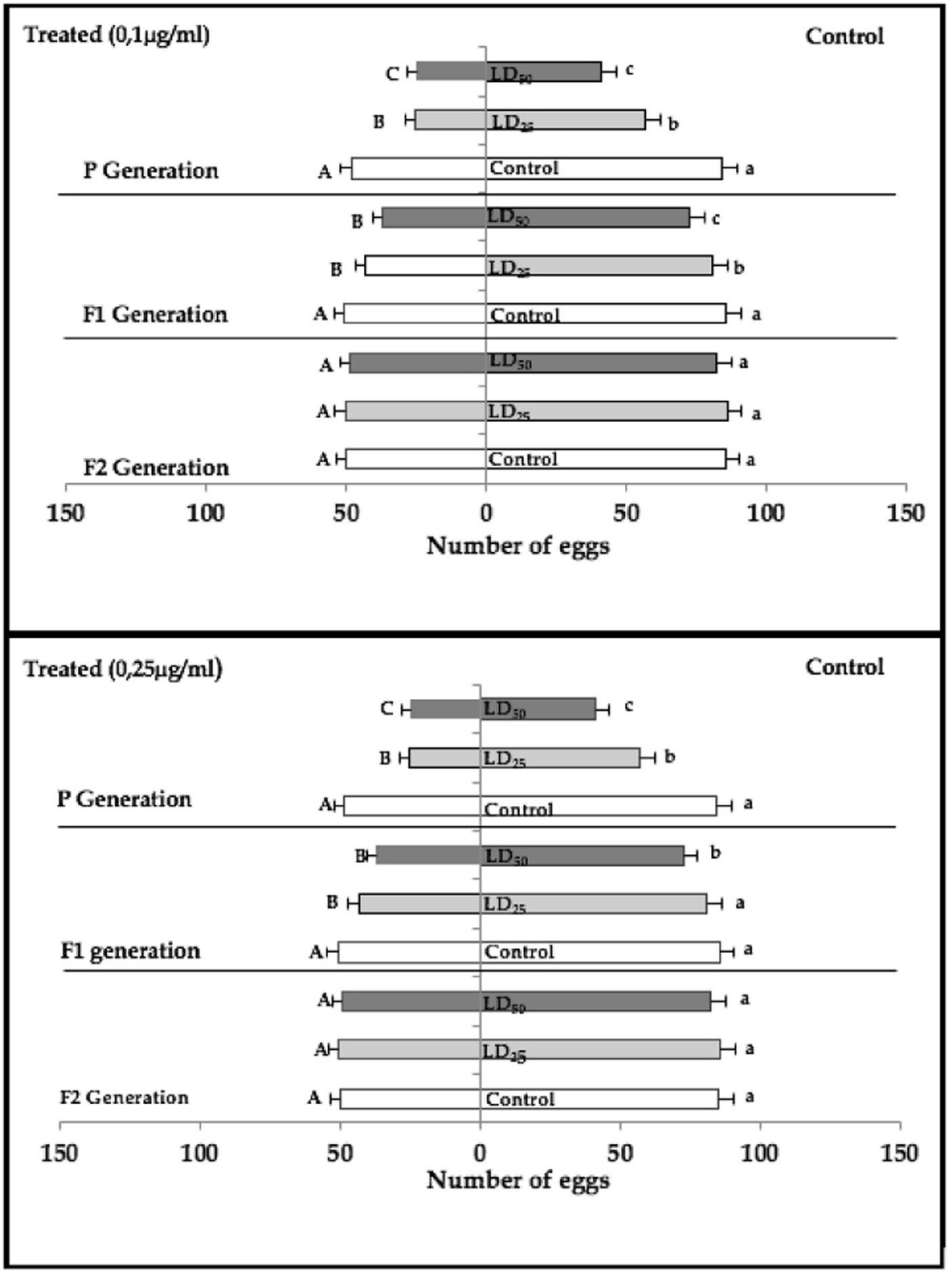
Egg-laying preference (m ± SE; n = 12 replicates) of female adults of *D. melanogaster* subjected to a free-choice test on food treated with azadirachtin at two doses (0.1 µg/ml and 0, 25 µg/ml). Different small letters indicate a significant difference between control and treated individuals of medium untreated and treated (P < 0.05). Different capital letters indicate a significant difference between individuals of the same dose in the different medium (P < 0.05).

The results concerning the dual choice experiments (Fig. 2) revealed an oviposition preference in control medium than in treated medium for all tested generations (P, F1 and F2). Furthermore, flies previously exposed to azadirachtin (early 3^rd^ instar larvae) showed a highest aversion to this substance compared to naïve flies and led fewer eggs for the two first generations (P and F1) with a more marked effects for parental generation (P < 0.001).

**Figure 3:**
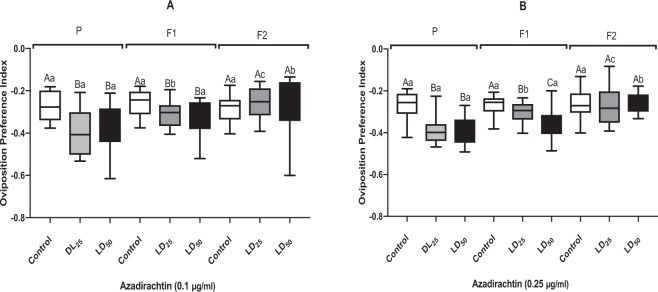
Oviposition preference index (m ± SE; n = 12 replicates) of female adults of *D. melanogaster* subjected to a free-choice test on food treated with azadirachtin at two doses (**A**: 0, 1 µg/ml; **B**: 0, 25 µg/ml). Different small letters indicate a significant difference between the same dose of different generations (P < 0.05). Different capital letters indicate a significant difference between difference tested doses of the same generation (P < 0.05).

The oviposition preference index (OPI) of adult females of *D. melanogaster* exposed, or not, to azadirachtin at larval stage of parental generation were always negative in all generations (Fig. 3).

In the generation P, statistical analysis showed significant differences between OPI of previously treated flies and controls flies with a dose-dependent response (Fig. 3). In addition, for medium 0.1 μg/ml, Mann-Whitney revealed significant effects between LD_25_ of the parental generation and the first generation (Mann-Whitney test U = 8; P < 0.001), LD_25_ of parental and second generation (Mann-Whitney test U = 20; P = 0.0018) but there was no difference between the first and the second generations (Mann-Whitney test U = 42; P = 0.0887). Similar results were observed for the LD_50_, with significant effects observed between the parental generation and the F1 (Mann-Whitney test U = 19; P = 0.0014), also between P and F2 (Mann-Whitney test U = 34; P = 0.0284) but no difference between F1 and F2 (Mann-Whitney test U = 58; P = 0.4428). For control, there was no difference between all tested generations.

Similar results were obtained for medium 0.25 μg/ml, Mann-Whitney test revealed significant effects between LD_25_ of the parental generation and the first generation (Mann-Whitney test U = 25; P = 0.0045), LD_25_ of parental and second generation (Mann-Whitney test U = 24; P = 0.0045) but there was no difference between the first and the second generations (Mann-Whitney test U = 66; P = 0.5512). For the LD_50_, significant effects were observed between the parental generation and the F2 (Mann-Whitney test U = 25.50; P = 0.0025), also between F1 and F2 (Mann-Whitney test U = 34; P = 0.0028) but no difference was observed between F1 and P (Mann-Whitney test U = 49; P = 0.1974). There was no difference between controls for all generations.

### Analyses of development

Results from development analysis of *D. melanogaster* are given in Tables 1 and 2, respectively for parental (exposed) and F1 (non-exposed) generation. Treatment of early third instar larvae at two tested doses (LD_25_ and LD_50_) decreased the number of larvae, pupae and the final number of organisms of parental generation with a dose-dependent relationship as expressed by the FNO which is always negative for the treated series. The development of F_1_ *D. melanogaster* doesn’t seem to be affecting by the early treatment of the parental generation. However, the FNO of tested flies (LD_25_, LD_50_ and control) in treated medium was significantly lower than in the control medium for both generations. There is no difference between the number of organisms reached the pupae stage and the final number of organism in both generations. In addition, treatment of early third instar larvae increased significantly (p < 0.001) the duration of larval and pupal development as expressed by T_50_, with dose-dependent manner only for the Parental generation (exposed) as compared to controls. There is no difference between the T_50_ of the tested flies in both treated and untreated medium.

**Table 1:** Effect of larval exposure to azadirachtin on development of parental generation (exposed) of *D. melanogaster*.

Concentration	Larvae			Pupae			Imagoes		
	N° of individuals	T_50_ (h)	Malformations (%)	N° of individuals	T_50_ (h)	Malformations (%)	N° of individuals	Malformations (%)	FNO
**Control Medium**									
Control	93.73 ± 1.31 A a	41.93 ± 0.25 A a	0.0 ± 0.0 A a	92.66 ± 1.41 A a	150.86 ± 1.43 A a	0.0 ± 0.0 A a	90.8 ± 1.48 A a	0.0 ± 0.0 A a	0
DL_25_	85.86 ± 1.22 A b	49.8 ± 0.63 A b	2.53 ± 0.96 A b	79.4 ± 1.37 A b	159.4 ± 0.35 A b	1.53 ± 0.70 A b	78.4 ± 1.52 A b	17.86 ± 2.65 A b	−13.41
DL_50_	80.20 ± 2.24 A b	60.93 ± 0.61 A c	3.6 ± 1.03 A b	75.06 ± 2.50 A b	166.2 ± 0.53 A c	4.4 ± 2.53 A c	73.2 ± 2.53 A c	20.33 ± 2.65 A b	−19,25
**Medium treated with azadirachtin 0.1 µg/ml**									
Control	89.93 ± 0.64 A a	42.06 ± 0.61 A a	0.0 ± 0.0 A a	86.26 ± 1 A a	151.46 ± 0.89 A a	0.0 ± 0.0 A a	81.60 ± 1.15 B a	0.0 ± 0.0 A a	0
DL_25_	79.93 ± 1.27 A b	49.6 ± 1.64 A b	4.06 ± 0.93 B b	69.4 ± 0.98 B b	160.13 ± 0.50 A b	4.20 ± 0.92 B b	67.4 ± 1.1 B b	15.86 ± 2.06 A b	−17.08
DL_50_	77.46 ± 1.52 A b	62.53 ± 1.68 A A c	7.6 ± 1.21 B c	66.93 ± 0.81 B b	167.06 ± 0.91 A c	3.73 ± 0.72 A b	65.66 ± 1.37 A c	16.13 ± 1.85 A b	−19.34
**Medium treated with azadirachtin 0.25 µg/ml**									
Control	80.13 ± 1.74 B a	43.86 ± 0.90 A a	0.0 ± 0.0 A a	74.8 ± 1.67 B a	150.8 ± 0.75 A a	0.0 ± 0.0 A a	73.53 ± 1.93 C a	0.0 ± 0.0 A a	0
DL_25_	72.53 ± 1.56 B b	54.13 ± 1.10 B b	7.06 ± 1.10 C b	63 ± 1.48 B b	159.06 ± 0.89 A b	9.06 ± 0.97 C b	60.64 ± 1.77 B b	19.4 ± 2.15 A b	−17.31
DL_50_	66.2 ± 2.18 B b	61.6 ± 0.98 A c	10.86 ± 0.91 B c	54.13 ± 1.85 C c	171.06 ± 0.69 A c	11.86 ± 1.07 B c	53.13 ± 1.82 B b	24.13 ± 1.76 B b	−26.94

Letters indicate a significant difference between the different tested doses of the same medium for each stage of development (P < 0.05). Different capital letters indicate a significant difference same doses tested of different medium (P < 0.05). (m ± SE; n = 15 replicates).

**Table 2:** Effect of larval exposure to azadirachtin on development of first generation (non-exposed) of *D. melanogaster*.

Concentration	Larvae			Pupae			Imagoes		
	N° of individuals	T50 (h)	Malformations (%)	N° of individuals	T50 (h)	Malformations (%)	N° final organisms	Malformations (%)	FNO
**Control Medium**									
Control	97.53 ± 0.80 A a	42.93 ± 0.46 A a	0.0 ± 0.0 A a	97.2 ± 0.80 A a	150.4 ± 1.22 A a	0.0 ± 0.0 A a	96.8 ± 0.76 A a	0.0 ± 0.0 A a	0
DL_25_	97.2 ± 0.82 A a	43.73 ± 0.85 A a	0.0 ± 0.0 A a	96.26 ± 0.94 A a	152.6 ± 1.05 A a	0.0 ± 0.0 A a	94.00 ± 0.95 A a	0.0 ± 0.0 A a	−2.85
DL_50_	97.73 ± 0.85 A a	42.93 ± 0.69 A a	0.0 ± 0.0 A a	96.13 ± 0.97 A a	152 ± 0.80 A a	0.0 ± 0.0 A a	93.93 ± 1.17 A a	0.0 ± 0.0 A a	−2.90
**Medium treated with azadirachtin 0.1 µg/ml**									
Control	89.62 ± 1.80 A a	43.06 ± 0.50 A a	0.0 ± 0.0 A a	87.86 ± 1.84 B a	150.86 ± 0.57 A a	0.0 ± 0.0 A a	85.60 ± 1.59 B a	0.0 ± 0.0 A a	0
DL_25_	84.2 ± 1.3 B a	42.6 ± 0.77 A a	0.0 ± 0.0 A a	82.4 ± 1.37 B a	152.73 ± 1.12 A a	0.0 ± 0.0 A a	82.51 ± 1.36 B a	0.0 ± 0.0 A a	−4.44
DL_50_	84.60 ± 1.32 B a	42.86 ± 0.79 A a	0.0 ± 0.0 A a	81.26 ± 1.48 B a	151.86 ± 1.19 A a	0.0 ± 0.0 A a	80.86 ± 1.57 B a	0.0 ± 0.0 A a	−5.30
**Medium treated with azadirachtin 0.25 µg/ml**									
Control	86.53 ± 1.97 A a	42.64 ± 0.83 A a	0.0 ± 0.0 A a	78.73 ± 2.17 B a	149.46 ± 0.94 A a	0.0 ± 0.0 A a	78.73 ± 2.17 B a	0.0 ± 0.0 A a	0
DL_25_	83.66 ± 1.73 B a	41.8 ± 0.82 A a	0.0 ± 0.0 A a	76.40 ± 1.23 B a	150.33 ± 0.71 A a	0.0 ± 0.0 A a	73.2 ± 1.48 C a	0.0 ± 0.0 A a	−6.41
DL_50‘_	78.8 ± 1.52 B b	42 ± 0.81 A a	0.0 ± 0.0 A a	76.86 ± 1.38 B a	151.4 ± 0.60 A a	0.0 ± 0.0 A a	73.73 ± 1.55 B a	0.0 ± 0.0 A a	−5.37

Letters indicate a significant difference between the different tested doses of the same medium for each stage of development (P < 0.05). Different capital letters indicate a significant difference same doses tested of different medium (P < 0.05). (m ± SE; n = 15 replicates).

**Figure 4:**
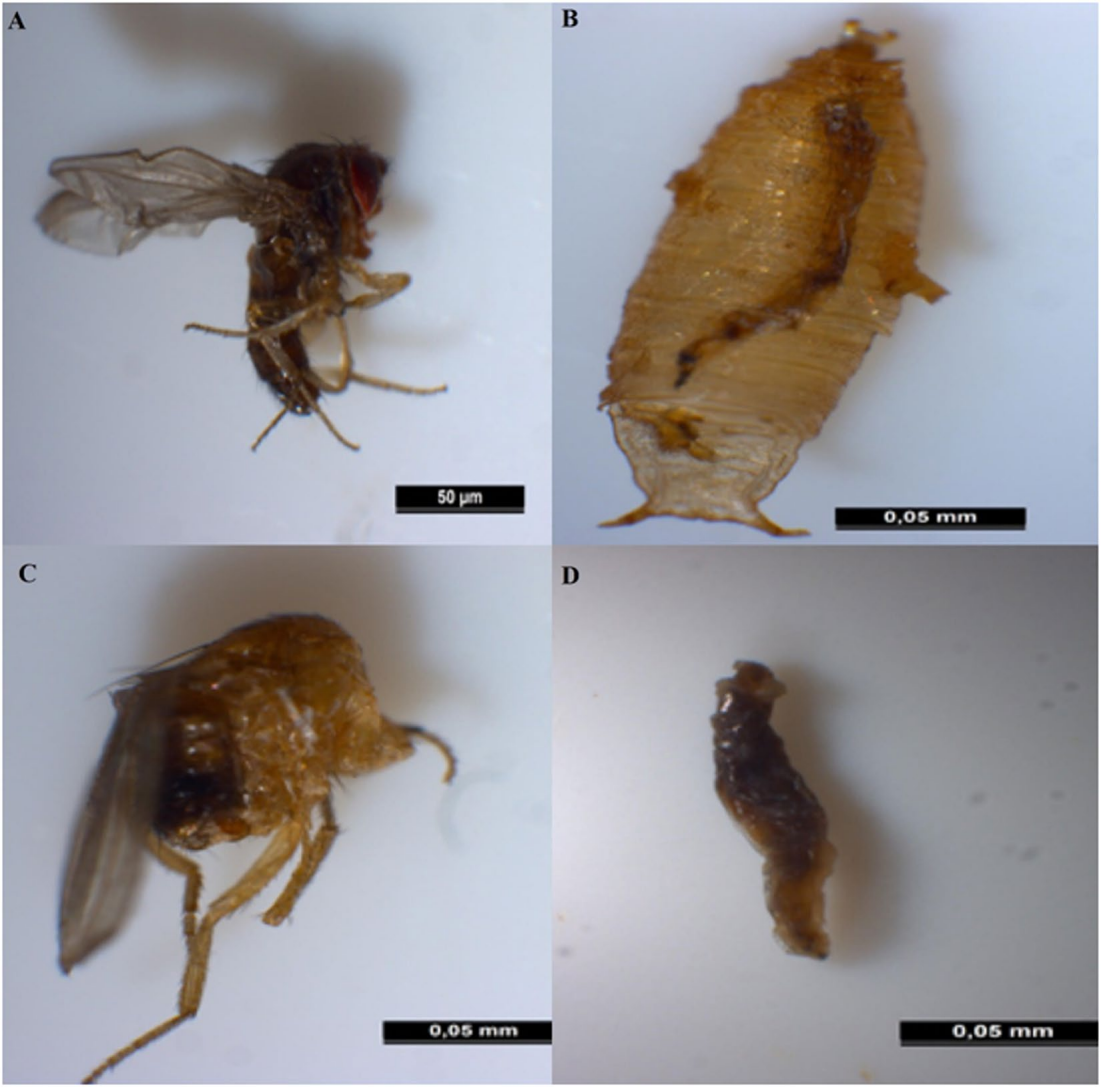
Examples of the most frequent malformations of *D. melanogaster* (n = 50). (**A**) Malformed abdomen and wings curved and smaller body shape; (**B**) dead adults inside pupae; (**C**) malformed adult; (**D**) burned larvae.

Larvae, pupae and imagoes of the parental generation showed several types of malformations and anomalies followed by death at each stage of development of *D. melanogaster*. The most prominent malformations detected are incomplete and malformed imagoes (malformed abdomen and wings), curved and smaller body shape, burned larvae, dead adults inside pupae (Fig. 4).

**Figure 5:**
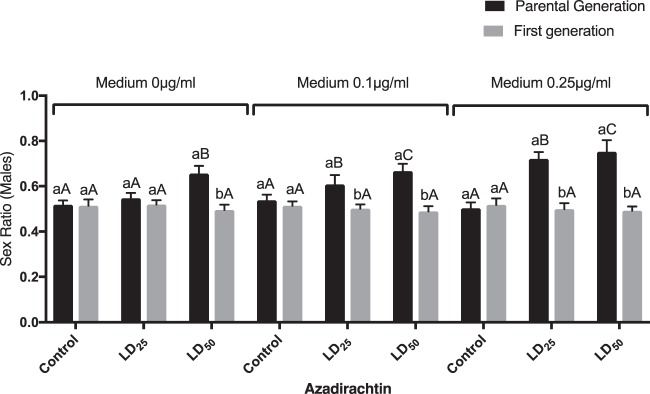
Effect of azadirachtin (LD_25_ and LD_50_), topically applied on early third instars larvae of *D. melanogaster* on sex ratio of adults emerged. Different small letters indicate a significant difference between generations of the same medium (P < 0.05). Capital letters indicate a significant difference between control and treated individuals of the same medium (P < 0.05). (m ± SE; n = 15 replicates).

Pre-imaginal exposure of azadirachtin induced a male-biased sex ratio only for the parental generation with a dose-dependent relationship (Fig. 5). Kruskal-Wallis test revealed significant effects between the different tested insect (Control, LD_25_ and LD_50_) in untreated medium (KW = 9.30; p = 0.0095), medium 0.1 μg/ml (KW = 8.02; p < 0.0181) and medium 0.25 μg/ml (KW = 18.85; p < 0.0001) for the parental generation.

### Survival analysis of adults

**Figure 6:**
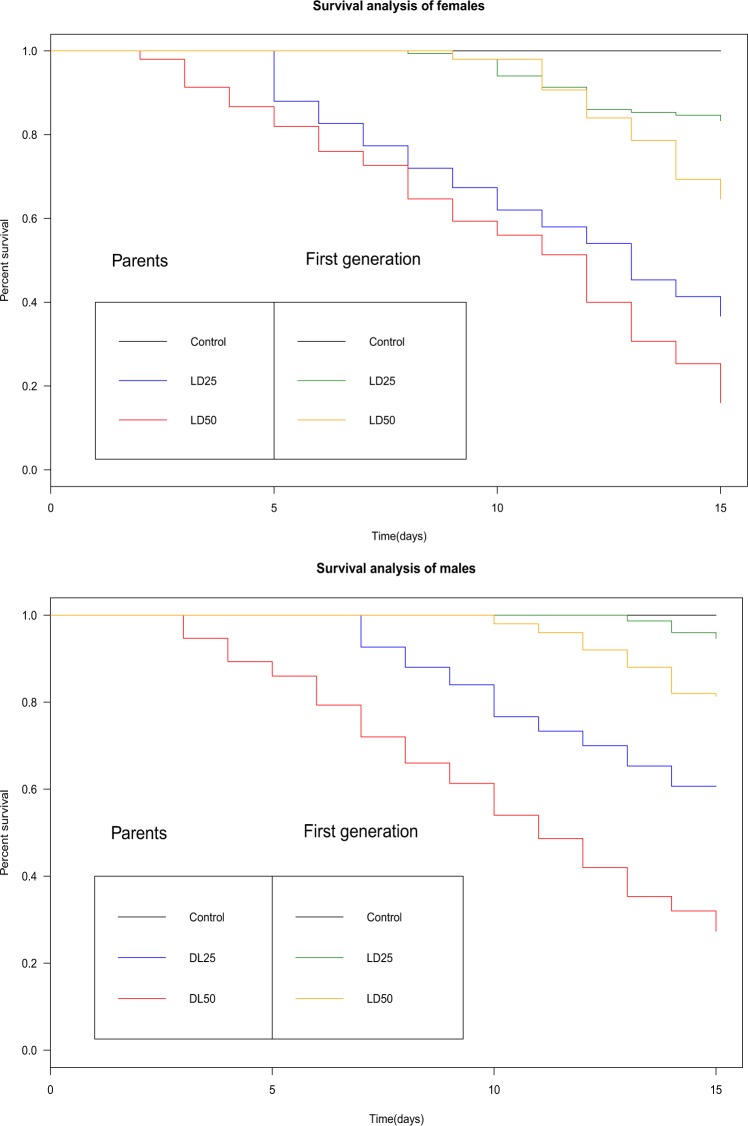
Effect of azadirachtin (LD_25_ and LD_50_), topically applied on early third-instar larvae of *D. melanogaster* on the adult’s survival (male and female) of two generations tested (p < 0.05).

A survival analyses during the 15 first days of adults previously treated with azadirachtin as 3^rd^ instars larvae (Fig. 6) revealed a rapid reduction of adult surviving of the generation P (Male: Kaplan-Meier test, χ2 = 184, df = 2, P < 0.001; Female: Kaplan-Meier test, χ2 = 214, df = 2, P < 0.001). Lower mortality was noted for the generation F1 compared to parental. (Male: Kaplan-Meier test, χ2 = 39.1, df = 2, P < 0.001; Female: Kaplan-Meier test, χ2 = 63.1, df = 2, P < 0,001). Flies mortality was dose-dependent and the females were more affected by the treatment.

For the control series no mortality was recorded for both tested generations. For the treated series, the lowest dose (LD_25_) decline the adult’s survival to 49% for males and 36% for females of the P generation versus 94% for males and 84% for females of the F1 generation. The highest dose (LD_50_) induced more marked effects on adult’s survival with 27% for males and 16% for females of the P generation and 81% for males and 64% in females for the F1 generation. Survival of 100% was noted for males and females of the F2 generation.

## Discussion

Azadirachtin’s impact on reproduction have been reported on different insect species^21,41–45^. Our study has demonstrated that a single azadirachtin treatment (LD_25_/LD_50_) of *D. melanogaster* larvae reduced eggs number affecting negatively the fecundity of surviving females, not only through direct sublethal effects in exposed individuals, but also through transgenerational effects on F1individuals that were never directly exposed to the insecticide.

Oviposition is a complex and critical activity in the life cycle of an insect with a variety of factors that influence both physiology and subsequent behavior, that lead to egg deposition by an insect which tries to ensure safety to their progeny. Reduced fecundity and fertility after azadirachtin treatment has been reported in many insects including *Spodoptera littoralis*, *D. melanogaster, Galleria mellonella, Dysdercus cingulatus, Tuta absoluta* and *Helicoverpa armigera*^17,41,43–46^ and could be correlated to the negative action of azadirachtin on yolk protein synthesis and/or its uptake into oocytes^21^.

Ecdysteroids, JH and insulin/insulin-like growth factor signalling (IIS) regulation are crucial for reproduction of *D. melanogaster*^47^. Vitellogenesis in females was stimulated under JH action and has led to oocytes development, JH synergic action with 20E and IIS controls the nutrient-sensitive checkpoint necessary for oocytes formation^47^. Consequently, reduced fecundity may be related to the antagonist action of azadirachtin on major hormones controlling the reproductive process (JH/ecdysteroids)^7^.

In *Anopheles stephensi*, azadirachtin treatment has led to abnormal ovaries structure with a complete arrest of oogenesis, vitellogenesis and vitelline envelope formation impairment, as well as follicle cells degeneration^48^. Ovaries of azadirachtin-treated females of *Heteracris littoralis* also showed complete shrinkage with suppressed oocyte growth^49^, in addition to mitochondria disintegration and follicular cells destruction^49^. Moreover, Azadirachtin reduced mating success in *D. melanogaster* flies and negatively affected cyst and oocyte number and size^45^. Its treatment also affected food intake and digestive enzyme activity in the midgut, in these species^10^. This may disturb oogenesis and vitellogenesis since ecdysone and JH rates are affected by nutrient availability, which acts as positive regulator on insulin pathway conferring ovaries the necessary signalling for a normal oogenesis^50,51^.

In addition, tested flies of all generations preferred the control medium for oviposition avoiding the azadirachtin ones for the two tested doses and conditions (no-choice and free choice). A low oviposition rate of non-exposed (naïve) flies in azadirachtin-treated areas could be due to the known repellent, deterrent, and locomotor stimulation effects of azadirachtin and other neem-based insecticides, which were reported by Silva *et al*.^52^ for medflies *Ceratitis capitata*. Valencia-Botín *et al*.^53^ also suggest that the repellent property of neem extracts is the major factor responsible for the reduction of eggs number in *Anastrepha ludens* (Loew)^53^. The ovipository behavior inhibition may have a valuable impact in pest control.

In addition, flies who have already been treated (third instar larvae of P generation) showed an increased aversion to azadirachtin in comparison to the naïf flies. This continued for two successive generations (P and F1). When oviposition sites were treated with azadirachtin or other neem-based compounds, oviposition repellency, deterrency, or inhibition occurred in several insect species that are able to detect the bioinsecticide on the treated surface^7,14,43,54,55^. The capacity of insects to retain memory from early life exposure affecting the adult response was reported^38,56–58^. In *D. melanogaster*, females avoided oviposition on sites containing azadirachtin after larval exposure to the bio-insecticide^7^.

Here, we have reported for the first time that the negative effects of a single larval exposure to azadirachtin can also be passed on to the F1 generation (transgenerational effects). Environmental toxicants such as insecticide are able to provoke epigenetic alterations, which can be inherited in the next generations^59^. This may explain the reduced fecundity and oviposition avoidance in the non-exposed generation (F1).

Our study has also demonstrated that azadirachtin applied during the third larval instar of parental generation (LD_25_ and LD_50_) negatively affected various life traits of *D. melanogaster*, in a dose-dependent manner, as it significantly reduced larval, pupation, and emergence rate of the exposed generation. The biopesticide also significantly prolonged the larval and pupation period of development inducing important delays in immature stages development and affect sex ratios (with fewer females in the offspring) of the same generation. Additionally, the treatment induced morphological alterations of larvae, pupae and adults only in the exposed generation (P generation). The most prominent abnormalities were burned larvae, larva-pupa intermediate, pupa-adult intermediate, deformed wings, smaller body size and deformed abdomen. The recorded malformations finally resulted in insect dead. Similar results were noted in *D. melanogaster*^37^, *Hyalomma anatolicum excavatum*^60^ and *Spodoptera litura*^22^. Finally, a decline in adult’s survival was noted for the two successive generations with more marked effects among the P generation.

Azadirachtin is known to reduce pupation and eclosion rates of many insects like *Aphis glycines*^61^, *Plodia interpunctella*^62^, *Aedes aegypti*^63^ and *D. melanogaster*^21^. A negative impact of azadirachtin on the immature stages was expected due to its insect’s growth disruptor (IGD) action by suppressing haemolymph ecdysteroid and JH peaks^25,64^. Furthermore, azadirachtin is known to cause nucleus degeneration in the different endocrine glands (prothoracic gland, *corpus allatum* and *corpus cardiacum*) controlling insects moulting and ecdysis, which could act as generalised disruptor of neuroendocrine system^24^. Azadirachtin alters the growth and molting process of several insects by compromising their survival^7,20,43,65,66^. Lai *et al*.^67^ reported that azadirachtin down regulated the expression of different genes that are linked to hormonal regulation. This could explain the developmental aberrations observed in our results. Azadirachtin is also known to affect *Drosophila* nutrient intake and metabolism compromising the nutritional signals, which result in a decrease in insect weights and growth rates, and thus resulting in smaller body size impacting survival^10,25,37,66^. The male biased sex ratio under azadirachtin treatment was reported in literature^67,68^.

In conclusion, the present study indicated that pre-imaginal exposure to sublethal doses of azadirachtin affects the fecundity, oviposition preference, and the survival of *D. melanogaster* of parent generation as well as the non exposed F1 generation. The treatment triggered life history traits variation in the P generation.

Results demonstrated that a single azadirachtin application significantly reduced the survival of flies over two successive generations (P: exposed and F1: unexposed) while insects showed clear recovery in the survival rates in the second generation (F2). These findings reflect a long term effects through developmental stage and generations. This effect is consistent in the two first generations could be considered as advantage for pest control by compensating the well-known fast degradation by sunlight and low persistence of azadirachtin in environment (half-life DT_50_: 1.7–25d)^23,69^ and suggest a possible reduction both in application frequency and total amount of pesticide used.

Furthermore, the decreased fecundity and survival in P and F1 generations indicated an absence of resurgence induction in offspring, even after full restoration in F2, when parental flies were treated. This translated an absence of hermetic effect, which is considered as a serious problem of exposure to sublethal doses in agriculture.

In addition, the treatment extended the aversive effect induced by azadirachtin to over two successive generations. This could contribute as push-pull strategies that increase its insecticidal effects in integrated pest management programs.

## Material and Methods

### Flies

Wild-type Canton-S strain of *D. melanogaster* flies were reared on artificial fly food (cornmeal/agar/yeast) at 25 °C, 70% humidity and 12D-12 L cycle^10^.

### Treatment

Neem Azal-TS (1% azadirachtin A, Trifolio-M GmbH, Lahnau, Germany) was solubilised in acetone for topical application (1 µl/larvae according to Bensebaa *et al*.^36^). The bioinsecticide was applied on *D. melanogaster* early third-instar larvae using two lethal doses of immature stages, 0.28 µg (LD_25_) and 0.67 µg (LD_50_)^37^_._ Controls received 1 µl acetone (solvent) and all flies were kept under the same conditions as cited above. All experiments were performed over two consecutive generations, the exposed (parental generation: P) and non-exposed (first generation: F1) generation.

### Fecundity and oviposition site preference

We assessed the egg-laying performances of the females of *D. melanogaster* using a no-choice test. Three mated females (3 days old) that were pre-exposed to azadirachtin at the larval stage (LD_25_ and LD_50_) were tested for 24 h in a petri dish (Ø = 65 mm) filled with 3 ml medium containing azadirachtin at two concentrations 0.1 and 0.25 μg/ml according to Bezzar-Bendjazia *et al*.^37^ in addition to acetone as control medium. These concentrations were not lethal with the short exposure time (24 h) used. At the end of the test, flies were removed, and the number of eggs laid on each medium was counted. The control medium was used to test the possible effect of azadirachtin on female fecundity. The experiment was repeated 12 times for each medium and each generation. Oviposition site preference was measured by means of dual choice experiments. Three fertilized females (3 days old) from controls and treated series (LD_25_ and LD_50_) were allowed to oviposit for 24 h in a free choice egg-laying device. This device consisted of a two petri dishes either filled with control medium (acetone) or treated medium (azadirachtin: 0.1, 0.25 μg/ml). After 24 h, the egg-laying preference was assessed by counting the number of eggs laid in each medium. The test was performed for two successive generations with 12 replicates for each medium and generation.

Oviposition preference index (OPI) defined as (number of eggs on azadirachtin medium – number of eggs on control medium)/total number of eggs was calculated^38^.

### Development assays

Ten controls or pre-exposed (LD_25_ and LD_50_) mated females (3 days old), named parental generation, were released into an oviposition box containing petri dishes filled with control (acetone) or treated medium (azadirachtin: 0.1 or 0.25 μg/ml) and left to lay eggs for 8 hours. At the end of the test, the flies were removed and a pool of 100 eggs for each experiment was transferred to a new petri dish containing the same medium. For all groups, we monitored the time course of larval development from egg to adult emergence by counting the number of third instar larvae, pupae, imagoes and their sex ratio, expressed as the number of males divided by the total number of emerged insects.

Next, ten parental flies from each condition (controls or treated) were crossed and the experiments were repeated for the non-exposed first generation (F1) as cited above with the same parameters recorded.

Furthermore, the developmental duration of each stage was recorded for the two tested generations expressed by T_50_ (time in hours, when 50% of population reached larval, pupal and imaginal developmental stage in vials). All insects were observed under stereo zoom microscope to find any morphological distortions and photographs were taken with Leica Z16 APO.

A factor describing the final number of organisms in comparison to control (FNO) according to Ventrella *et al*.^39^ was determined to compare the results:

$$FNO=\frac{{\rm{T}}-{\rm{C}}}{{\rm{C}}}\,\times 100$$

T = final number of organisms counted in treated medium.

C = final number of organisms counted in control medium.

Positive values of FNO show that number of organisms was higher in tested groups than within control, negative values mean that the number of individuals was higher in control than in exposed groups.

### Survival analysis of adults

Survival analysis was performed according to Linford *et al*.^40^. For each generation (P: exposed (LD_25_ and LD_50_), F1: non-exposed) newly emerged adults were sexed and housed separately into a plastic vials (15 flies per vial) containing fresh food. Insects were transferred to new vial every 2 days. The flies were kept under observation for 15 days during which mortality was assessed every 24 h. Ten replicates were done for each dose and generation.

### Statistical analyses

Data analysis was performed by R studio version 3.5.0 for Mac OS. The results were expressed as the means ± SE for each series of experiments. The homogeneity of variances was checked using Bartlett’s test. The Shapiro-Wilk statistic test was used for testing the normality.

Data from egg-laying preference and oviposition index preference was subjected to Kruskal–Wallis test and pairwise multiple comparisons using Dunn’s method. Development test were analysed with ANOVA followed by a post-hoc HSD Tukey test. Sex ratio was analysed using Kruskal–Wallis test and the FNO was calculated and shown. The results of the survival analysis were subjected to Kaplan–Meier survival test.
